# Sustainable Design and Evaluation of Children’s Food Packaging from the Perspective of Buyers’ Preferences

**DOI:** 10.3390/foods13233895

**Published:** 2024-12-03

**Authors:** Ying Xiao, Yihan Wang, Yangyang Wei

**Affiliations:** 1 Architecture and Design College, Nanchang University, Nanchang 330031, China; 2 School of Art, Wuhan Business University, Wuhan 430056, China

**Keywords:** consumer behavior, CRITIC–MABAC model, children’s food, packaging, sustainable design

## Abstract

Consumer behavior is one of the key factors influencing product sales, especially in food packaging design, where green, organic, sustainable, and human-centered designs are more effective in promoting food sales. This paper aims to develop a sustainability evaluation method for children’s food packaging. The study first explores the theoretical foundations of sustainability, establishing a systematic set of quantitative indicators and evaluation criteria. Based on this framework, the research gathers consumption behavior, rating data from 250 parents of various ages, professions, and income backgrounds. Using the CRITIC model, the study performs dimensionless processing and detailed quantitative evaluation of the indicators’ comparability, contradictions, and information content to allocate weights for the sustainability evaluation metrics. Furthermore, the MABAC model is applied to construct a weighted decision matrix and boundary approximation area, ranking the sustainability of 20 representative children’s food packaging design schemes (S1–S20). The results show that Scheme S1, after calculation using the CRITIC–MABAC model, has a total distance of 0.214 from the boundary approximation area, exhibiting the smallest deviation from the ideal solution across multiple evaluation criteria and achieving the best overall performance. Building on the optimal Scheme S1, this study comprehensively considers key elements such as eco-friendliness, safety, functionality, and educational value in the optimization of a sustainable design for children’s fruit puree packaging. The research validates the practicality and effectiveness of the quantitative model through the sustainable design and evaluation of children’s food packaging from a consumer behavior perspective, promoting sustainability design and optimization in the children’s food packaging sector.

## 1. Introduction

With the growing severity of global environmental pollution and society’s continuous pursuit of sustainable development, the field of children’s food is facing unprecedented responsibilities and challenges. Children are the most vulnerable group in society, and so their food’s safety and their health are directly linked to the future of humanity and the development of society [1]. Therefore, the sustainable design evaluation and optimization of children’s food packaging not only reflects social responsibility but also represents a deep concern for the shared future of humanity.

The design and development of children’s food packaging is an evolving field, with its history tracing back to the single-functional packaging designs aimed at protecting food. Today, it has evolved into a complex design system that considers multiple dimensions such as health, safety, and environmental sustainability. Early research on food packaging mainly focused on the physical protective properties of packaging materials and how to extend food shelf life. With the rise in environmental awareness, scholars have begun to pay attention to the environmental impact of packaging materials, exploring the use of recyclable or biodegradable materials to reduce environmental pollution. In the field of children’s food packaging design, biopolymers such as polylactic acid (PLA) are among the more common packaging materials used for children’s food [2].

On the other hand, Marloes de Brabandere’s research focuses on children’s reactions to sustainability labels on food packaging. They found that children’s level of environmental awareness is an important factor influencing the effectiveness of sustainability labels [3]. For children with low environmental awareness, combining sustainability labels with visual cues can enhance their evaluation of packaging and increase purchase intentions. For children with higher environmental awareness, visual cues may weaken the effectiveness of text labels. This finding provides strategic guidance for food manufacturers and marketers, suggesting that packaging labels should be designed according to children’s level of environmental awareness.

Although various methods and frameworks for evaluating the sustainability of food packaging have been proposed, such as the Life Cycle Assessment, the Sustainable Food Packaging Design Framework, and the SPA-based Packaging Sustainability Assessment Tool [4], these methods often fail to fully integrate sustainability, especially in terms of material safety and environmental impact. Furthermore, these evaluation models often lack practical application validation, limiting their applicability. It is evident that while some progress has been made in environmental awareness and packaging design in children’s food packaging, there are still limitations in sustainable evaluation methods. In the context of sustainable design, the function of children’s food packaging has gone beyond traditional practicality and has expanded to environmental friendliness. Therefore, constructing a comprehensive sustainability evaluation system requires the consideration of multiple dimensions of children’s food packaging. For example, factors such as the packaging’s environmental impact and whether it fulfills its role in protecting the product must be considered. Additionally, children’s physiological characteristics and consumer psychology should be taken into account, ensuring the packaging is safe, convenient, fun, and environmentally friendly [5].

To address the above limitations, this study introduces the CRITIC–MABAC model, aiming to comprehensively assess the sustainability of children’s food packaging solutions through a multi-attribute evaluation method based on consumer behavior. This model integrates key dimensions such as environmental friendliness, economic efficiency, and safety, with a particular emphasis on children’s health factors. Through this systematic evaluation method, the study seeks to promote the children’s food packaging industry towards a more environmentally friendly, safe, and user-friendly direction. The research combines case studies on the optimization of children’s food packaging design, actively exploring sustainable design and optimization of children’s food packaging from the perspective of consumer behavior.

## 2. Study on the Sustainability Status and Evaluation System of Children’s Food Packaging

### 2.1. Sustainability Status of Children’s Food Packaging

In exploring the trends of sustainable development in children’s food packaging, meeting the dual demands of environmental protection and buyers has become a focal point for researchers. Studies in materials science, consumer behavior, policy development, and market dynamics all contribute to the sustainability of children’s food packaging.

In terms of material innovation, biodegradable and edible packaging materials are becoming hotspots for research and application. Cellulose-based hydrogel films, known for their high mechanical strength and biodegradability, have been widely applied in food packaging [6]. Multilayer bio-based films are also gaining attention for their excellent mechanical properties and barrier performance. These novel materials not only help reduce environmental pollution but also improve food quality and safety [7]. Erik Pauer et al. highlighted that food loss, waste, and recycling rates related to food packaging are key aspects in assessing environmental sustainability. Material selection should consider resource consumption and carbon footprint, prioritizing biodegradable materials to reduce the risk of long-term environmental pollution [4]. Notably, according to the research of Urška Vrabič-Brodnjak and Iva Jestratijević, 61% of brands have adopted recyclable, refillable, or bio-based alternatives, yet 39% of brands still do not disclose details of their packaging materials [8]. This indicates that the concept of sustainable packaging design and its evaluation systems need further development.

In terms of consumer education, studies have shown that conveying ecological information through packaging design is an effective way to improve children’s environmental awareness. In the Brazilian market, ecological information has been integrated into packaging designs targeted at children to promote sustainable education. However, despite most children being able to correctly answer questions related to waste disposal, they still struggle to identify the appropriate recycling bins. This underscores the need to further enhance children’s education and practical knowledge about sustainable packaging [9]. Juliana de et al. also pointed out that encouraging children to read food labels helps them gain more nutritional knowledge, increases the frequency of reading nutritional labels on industrialized products, and improves their understanding of food [10]. Furthermore, the Journal of Marketing Management revealed that children’s attention to sustainable diets can be enhanced through the effective combination of visual cues and verbal labels [11]. Therefore, children’s packaging design should consider how to effectively convey environmental messages and inspire children’s interest in sustainability.

In the context of regulations, global legislative trends on sustainable food packaging are continuously strengthening. Regions such as Europe, India, South Korea, Japan, China, Australia, the United Kingdom, and the United States are enacting policies to reduce waste and promote a circular economy [12]. These regulations are driving the development of new eco-friendly packaging materials and encouraging companies to adopt greener packaging methods. Additionally, Elford’s research was the first to explore environmental sustainability practices in food provision within Australian childcare services. Their preliminary survey results showed that most childcare services have incorporated environmental sustainability into their policies, highlighting the potential of policies in promoting sustainable food practices [13]. In terms of user needs, Victoria Norton’s study found that 74% of consumers are willing to pay a premium for sustainable packaging, indicating a strong market demand for sustainability [14]. This emphasizes the importance of considering the environmental and sustainable attributes of packaging in children’s food design. A. Kurtz and R. Thomopoulos focused on consumer behavior, particularly parents’ concerns about the safety and sustainability of baby food. Their large-scale survey in France analyzed consumer priorities and risk perceptions when selecting baby food. The study found that health factors, such as the absence of harmful substances and nutritional balance, were the top concerns, far outweighing environmental and price factors. Additionally, chemical contaminants and the safety of packaging materials were the most worrying issues for consumers [15]. Alazne Arraztio Cordoba emphasized that safety is a critical element in the design of baby and children’s food packaging, and packaging elements significantly influence children’s food choices and intake [16]. D. Folinas et al. noted that safety and protection are major concerns for parents, with designs such as pull tabs and tamper-evident seals reducing potential risks for children when unsupervised [17].

As global awareness of environmental protection increases, the sustainability of children’s food packaging has become a research hotspot [18]. This includes the use of eco-friendly materials to minimize environmental impact and leveraging packaging as a tool to educate children about the importance of sustainability. Furthermore, the strengthening of global legislative trends and the growing acceptance of sustainable packaging in the market, especially among younger and environmentally conscious consumers, are further driving advancements in this field [19].

### 2.2. Study on Sustainability Evaluation Systems

The sustainability evaluation system is a multidimensional and multi-tiered framework designed to comprehensively assess and promote economic, social, and environmental sustainability. Julia Frojan et al. proposed that the design and evaluation of children’s food packaging is a complex and multifaceted process, encompassing dimensions such as environmental protection, food safety, and social value [20]. The construction of a sustainability evaluation system involves theoretical framework development, indicator system design, and the selection of evaluation methods, all requiring a scientifically sound framework [21]. For instance, the theoretical framework for regional sustainability evaluation emphasizes the relationship between social, economic, and population development goals and the quality of the natural environment [22]. Additionally, sustainability evaluation models suggest systematic analysis principles from economic, social, demographic, resource, and environmental perspectives [23].

The principles and theoretical foundations of sustainability evaluation are also critical discussion points. Schmidt-Scheele et al. emphasized the importance of considering citizens’ needs regarding sustainability indicators [24]. Other studies have introduced new theoretical foundations for sustainability, categorized into three normative and functional categories: system integrity, justice, and quality of life [25]. The theoretical foundation of the sustainability evaluation system constructed in this study is derived from an in-depth understanding of the packaging design domain, including the 4R1D principle, national environmental guidelines, and consumer surveys. This study adopts the 4R1D principle—Reduce, Reuse, Recycle, Remanufacture, and Degradable—as a guiding framework for establishing ecological indicators in packaging design. The evaluation system strictly adheres to national environmental standards to ensure consistency with current regulations and industry practices. Data collected from surveys of children’s food packaging buyers reflect the preferences and needs of actual consumers who have purchased children’s products, providing empirical support for the evaluation system.

The establishment of sustainability evaluation indicators and standards delves into key aspects such as the selection of evaluation methods, requirements for indicator system construction, and the application of theoretical foundations and principles [26]. This study employs the indicator listing method to construct an environmental indicator system, comprehensively considering five dimensions: ecological sustainability, safety, functionality, educational value, and economic efficiency. These dimensions collectively assess the sustainability of children’s food packaging. The study leverages the theoretical frameworks of the 4R1D principle, national environmental standards, and survey data to provide robust theoretical support for the evaluation system. In practical evaluation processes, this system is integrated with the CRITIC–MABAC evaluation method to enhance the scientific rigor of the assessment. This approach offers a novel evaluation methodology aimed at advancing the sustainability goals of children’s food packaging and fostering the healthy development of the children’s food sector.

## 3. Materials and Methods

### 3.1. Research Subjects and Approach

In the empirical analysis phase, the study selected 20 representative children’s food packaging design schemes available on the market (S1–S20) for in-depth analysis using the CRITIC–MABAC model. Due to the evaluation of advantages and disadvantages of commercially available products, this study cannot fully disclose the detailed information of all 20 children’s food packaging schemes. However, the research findings of the evaluation methods are described in a way that ensures a balance between maintaining research transparency and adhering to confidentiality obligations. The analysis covered aspects such as the selection of environmentally friendly materials, material safety, and packaging freshness protection, aiming to comprehensively evaluate the sustainability of children’s food packaging from the consumer’s perspective and to verify the effectiveness and applicability of the model.

#### 3.1.1. Research Subjects

The study selected 20 children’s food packaging design cases (S1–S20) available on the market. Using the CRITIC–MABAC model and considering key sustainability indicators such as eco-friendliness, safety, functionality, educational value, and economic efficiency, the study identified the optimal design scheme. The selected optimal design achieved a balance and coordination across multiple dimensions of sustainability, demonstrating the unification and comprehensiveness of sustainable principles. The five key evaluation indicators are as follows:

Eco-friendliness Evaluation Indicator: The study analyzed the use of environmentally friendly materials in children’s food packaging samples, the implementation of minimalistic and reduced packaging design, and their environmental impact throughout their lifecycle. Factors considered include the selection of eco-friendly materials, the greenness of the production process, and the recyclability or biodegradability of the packaging post-use.

Safety Evaluation Indicator: This indicator focuses on whether children’s food packaging materials are non-toxic and safe, as well as whether the packaging incorporates child protection designs, such as easy-to-operate opening mechanisms and leak-proof features, aimed at reducing the risk of injury to children during use.

Functionality Evaluation Indicator: This primarily assesses the ease of use, convenience, and freshness and protection performance of children’s food packaging, which directly impacts consumer experience and the preservation of food freshness. The study analyzed the portability, storage convenience, and protective effect of the packaging on the food. Research by N.N. Minina and O. Sinelnikova indicates that effective packaging design not only protects food from negative environmental impacts but also prevents the rapid spoilage of products [27].

Educational Value Evaluation Indicator: This includes evaluating the integration of environmental education elements and interactive learning features in the packaging, and assessing the packaging’s ability to convey environmental information, provide opportunities for interactive learning, and inspire children’s interest in sustainability.

Economic Efficiency and Market Competitiveness: Economic efficiency and market competitiveness are also important dimensions of the evaluation. The study analyzed the cost-effectiveness and market appeal of each packaging design to ensure that the selected solutions are not only environmentally friendly but also marketable.

#### 3.1.2. Research Roadmap

**Figure 1:**
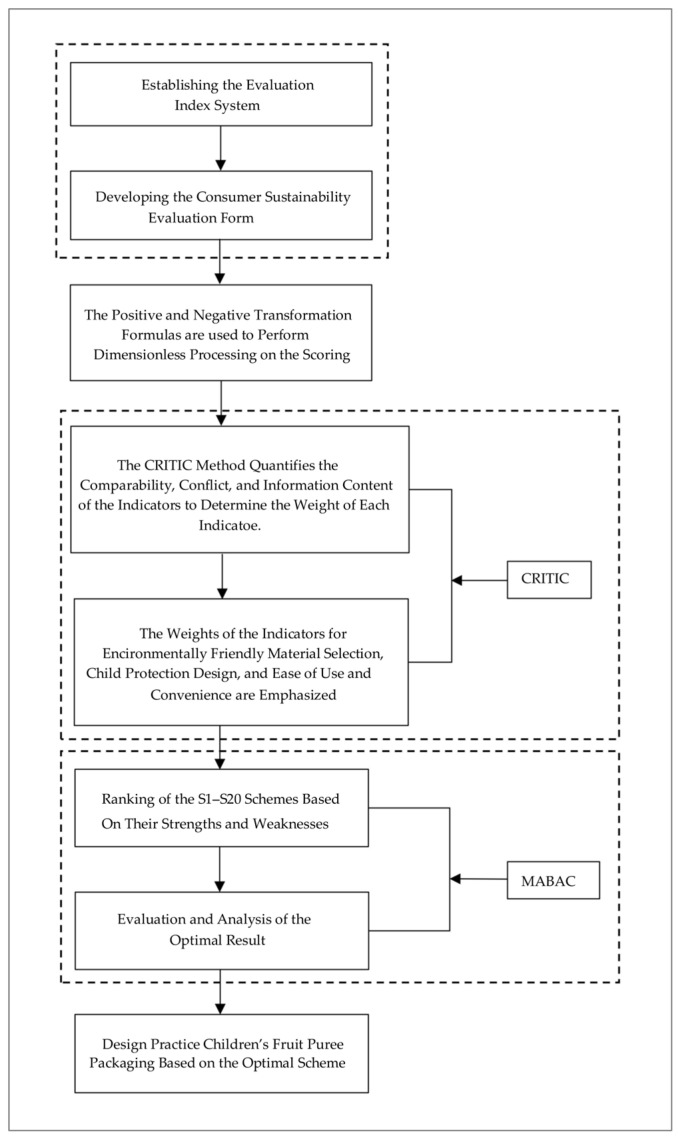
Research Technical Roadmap.

The research begins with a theoretical foundation, including principles such as the 4R1D principle and national environmental standards. Furthermore, a survey was conducted to collect consumer awareness data from 250 parents of children, which formed the foundational data for the evaluation index system. Through an in-depth analysis of the concept of sustainability and dialogs with consumers, the study established an evaluation system consisting of five primary evaluation indicators and eleven secondary indicators. This system encompasses key dimensions such as eco-friendliness, safety, functionality, educational value, and economic efficiency to ensure the comprehensiveness of the evaluation process, as illustrated in Figure 1.

After establishing the evaluation index system, the study applied positive and negative transformation formulas to standardize the collected data, aligning data from different individuals and characteristics to a uniform measurement scale. This ensures that all indicators can be compared and evaluated on the same scale during the multi-dimensional evaluation process, avoiding data analysis biases that may arise from differences in data units or magnitudes. Based on the standardized data, the study employed the CRITIC model to quantify the evaluation indicators. This model allocates weights to the indicators by comprehensively considering their contrast intensity, conflicts, and information content. The CRITIC model assesses the relative importance of each indicator based on its contrast, conflict, and information content [28], accurately identifying the core indicators in children’s food packaging design. This approach not only reveals the importance ranking of the indicators but also provides a deeper understanding of the role and impact of different indicators within the overall design [29,30].

After determining the indicator weights using the CRITIC model, the study further applied the MABAC model to conduct a detailed ranking analysis of the sustainability schemes for children’s food packaging design. As an efficient multi-criteria decision analysis tool, the MABAC model enables a comprehensive evaluation of various schemes based on their performance across different evaluation indicators [31]. The application of the MABAC model allows for an analysis of the relative strengths and weaknesses of different design schemes, ensuring transparency and rationality in the decision-making process [32].

### 3.2. Construction of the Sustainability Indicator System for Children’s Food Packaging Design

#### 3.2.1. Sustainability Evaluation Dimensions for Children’s Food Packaging Design

Erik Pauer, Bernhard Wohner, and others proposed the 4R1D principle of sustainable packaging design: Reduce, Reuse, Recycle, Renewable, and Degradable. This framework provides an ecological evaluation indicator, ensuring that sustainability principles are implemented throughout key stages such as material selection, production processes, transportation, and recycling [4]. This indicator strictly adheres to national environmental standards, aiming to limit excessive packaging and ensure that children’s food packaging design fulfills its basic functions of protecting food and appealing presentation, while effectively avoiding unnecessary material waste and reducing environmental impact [33]. In the critical stage of material selection, there is a particular emphasis on the use of environmentally friendly materials, including biodegradable or recyclable options, while also considering the environmental impact of the material sources and their production processes. The goal is to achieve a harmonious balance between environmental friendliness and practicality through careful selection of eco-friendly materials, demonstrating responsibility for environmental protection and a strong focus on children’s food health and safety. This study adopts and references this comprehensive approach to ensure that children’s food packaging design not only aligns with sustainable development principles in theory but also achieves the goals of environmental protection and resource conservation in practice.

**Figure 2:**
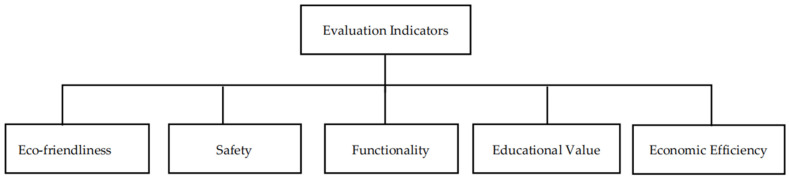
Sustainability Evaluation Indicators for Children’s Food Packaging Design.

Through a questionnaire survey, we identified several core consumer demands from parents regarding children’s food packaging design, including packaging safety, environmental friendliness, and transparency. These factors form the core considerations in establishing the sustainability evaluation indicators. Based on this, the study constructed a targeted and comprehensive evaluation system. This evaluation system is meticulously divided into five key indicators: eco-friendliness, safety, functionality, educational value, and economic efficiency (Figure 2).

Eco-friendliness is the primary dimension in the evaluation system, emphasizing the minimization of environmental impact throughout the entire lifecycle of packaging design. This involves the selection of environmentally friendly materials, prioritizing those that are recyclable, biodegradable, or sourced from sustainable resources [34]. Additionally, minimalistic and reduced packaging design is crucial, requiring the reduction in material usage as much as possible while still fulfilling basic functions, thereby lessening the burden on the environment. The assessment also considers the environmental impact during production, usage, and disposal processes, ensuring adherence to eco-design principles.

Safety is an indispensable part of the evaluation system, ensuring that packaging materials and design pose no harm to children. Material safety is fundamental, with the need to rigorously select non-toxic, harmless materials to guarantee they do not threaten children’s health [35]. The child protection design also takes into account the packaging’s structure and functionality, aiming to prevent potential hazards such as choking or cuts during use.

The functionality dimension focuses on the practicality and user experience of the packaging. Ease of use and convenience are core aspects, emphasizing designs that are easy to open, carry, and use while ensuring they effectively protect the food and extend its shelf life. Freshness and protective performance are also key components of functionality, requiring scientific design to ensure that the packaging provides an appropriate preservation environment, preventing spoilage and contamination.

The educational dimension views packaging design as a medium for conveying knowledge and values. By integrating environmental education elements into the packaging, it aims to raise children’s awareness of environmental protection. Additionally, interactive and educational features in the design encourage children to engage in environmental practices, fostering a sense of responsibility and practical skills.

Economic efficiency is also an important aspect of the evaluation system. It involves cost-effectiveness analysis and enhancing market competitiveness, ensuring that the design is economically feasible while maintaining competitiveness in the market. This not only includes controlling material and production costs but also considers the design’s impact on brand image and consumer choice [36].

#### 3.2.2. Sustainability Evaluation Indicator System for Children’s Food Packaging Design

**Figure 3:**
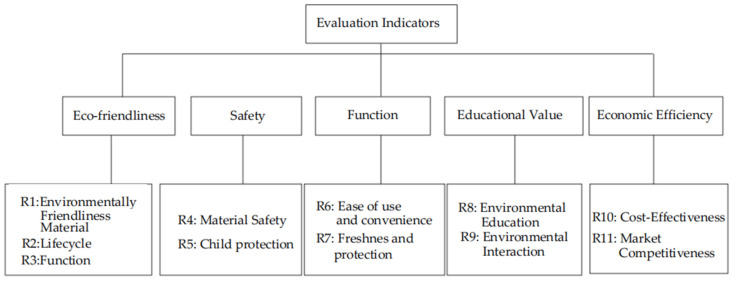
Sustainability Evaluation System for Children’s Food Packaging Design Schemes.

After establishing the five core primary evaluation indicators for the sustainability of children’s food packaging, the system was further refined based on feedback from consumer questionnaires by adding 11 more detailed secondary evaluation indicators. These 11 secondary indicators include: environmentally friendly material selection (R1), minimalist design (R2), lifecycle impact (R3), material safety (R4), child protection design (R5), ease of use and convenience (R6), freshness and protective performance (R7), environmental education elements (R8), interactive educational function (R9), cost-effectiveness (R10), and market competitiveness (R11). Together, the five primary indicators and the 11 secondary indicators form the sustainability evaluation system for children’s food packaging design, providing a powerful assessment tool for the sustainable development of children’s food packaging, as shown in Figure 3.

It is worth noting that this evaluation system is a multi-level evaluation framework, consisting of primary criteria and corresponding secondary criteria. Each secondary criterion is a further refinement and quantification of the primary criteria, as shown in Table 1. The tiered structure of the evaluation system ensures the rigor and scientific validity of the evaluation process.

Eco-friendliness, as the primary criterion in the evaluation system, holds a central position. It encompasses key aspects such as environmentally friendly material selection (R1), minimalist and reduced packaging design (R2), and lifecycle environmental impact (R3). First, environmentally friendly material selection (R1) emphasizes the prioritization of recyclable or biodegradable materials during the early stages of design. This not only helps reduce the burden on the environment but also actively promotes the circular economy. This viewpoint is supported by several studies, such as Houda Bouyarmane’s research, which highlights the importance of conducting environmental assessments in the early stages of product design by establishing databases on raw materials and manufacturing processes [37]. Minimalist and reduced packaging design (R2) advocates for streamlining the packaging design process to minimize unnecessary material use, thereby reducing resource consumption and waste generation. Lifecycle environmental impact (R3) requires a comprehensive assessment of the environmental effects of the packaging from production to final disposal, ensuring that the design reflects environmental friendliness at every stage. Haokun Tian noted that combining eco-risk assessment and lifecycle assessment methods during the early stages of product design contributes to sustainable design [38].

The safety criteria (R4 and R5) form a robust safeguard for protecting children’s health and safety. Material safety (R4) ensures that the materials selected are non-toxic and harmless, meeting or exceeding safety standards for children’s products to prevent any potential risks posed by the materials. According to existing regulations and guidelines, such as EU Regulation (EU) No 10/2011 and (EU) No 2018/213, it is important to evaluate whether the materials contain harmful chemicals such as bisphenol A (BPA). These regulations limit or prohibit the use of certain chemicals in specific types of food packaging [39]. Child protection design (R5) focuses on the safety of the packaging’s structure, requiring the incorporation of child-specific protective features. This includes avoiding sharp edges, ensuring that the packaging is easy for adults to open but difficult for children to open on their own, and avoiding small components that children could swallow. These design considerations aim to prevent a variety of potential safety hazards that children may encounter while using the packaging.

The functionality criteria (R6 and R7) focus on improving the practicality and user experience of the packaging. Ease of use and convenience (R6) are primary considerations in packaging design, ensuring that both children and parents can easily use the packaging. Whether it involves opening, resealing, or carrying the packaging, the design should provide a smooth and intuitive experience. Freshness and protective performance (R7) are crucial for ensuring food quality and safety. Carefully designed packaging must effectively maintain the freshness and nutritional value of the food while protecting it from environmental factors such as humidity, oxygen, and other potential contaminants. Optimizing this performance helps extend the shelf life of the food, ensuring that children can enjoy fresh, healthy meals while reducing waste from food spoilage.

The educational criteria (R8 and R9) emphasize the incorporation of environmental education elements (R8) into children’s food packaging design. The goal is to raise children’s awareness of the importance of environmental protection and cultivate their green consumption habits [40]. As noted by Khloud Ahmed and Wessam Amir, interactive packaging designs, such as foldable paper or scratch-off sections, can enhance children’s interaction with the packaging while conveying environmental concepts [41].

The introduction of interactive educational features (R9) adds dynamic educational value to the packaging design. Through interactive games, fun information, or thought-provoking questions, the packaging encourages children to actively participate in environmental practices, deepening their understanding of healthy eating and environmental protection. Children’s environmental behaviors are influenced not only by personal factors but also by family and social environments [42]. Therefore, discussing the environmental information on packaging and practicing environmental behaviors in daily life can strengthen children’s environmental awareness and involvement.

The economic criteria (R10 and R11) form an important part of the evaluation system. Cost-effectiveness analysis (R10), as the core component of this criterion, focuses on assessing the economic feasibility of the design solution, including strict control of production costs to ensure that the design is economically efficient. It also involves sharp insight into market trends and accurate forecasting to ensure that the packaging meets consumer needs while maintaining competitiveness in a crowded market. The enhancement of market competitiveness (R11) further expands the scope of the economic criteria, requiring the design not only to be cost-effective but also to demonstrate unique competitiveness in areas such as market positioning, brand image, and consumer preferences. This means that the packaging design must attract consumers with its price, but more importantly, win market recognition through quality, design innovation, and market adaptability.

#### 3.2.3. Quantitative Scoring Setup for the Sample Population

In the quantitative scoring phase of the original data, based on the principle of voluntary participation, the study collected questionnaire responses from 275 parents of children aged 0–12. Among these, 25 questionnaires were incomplete, resulting in 250 valid responses, with an effective response rate of 90.91%. To ensure the authenticity and reliability of the questionnaire data, the study required that these parents be the primary consumers of children’s food and have had multiple experiences purchasing children’s food. Based on the characteristics of the sample population reflected in the questionnaire responses, the sample was further categorized by different age groups, professional backgrounds, and income levels, as detailed in Table 2. The main content of the questionnaire involved having the respondents evaluate the sustainability of 20 different children’s food packaging designs (S1–S20) using the 11 indicators from the children’s food packaging sustainability design evaluation system. A 9-point rating scale (Table 3) was used for this evaluation to generate the sustainability score table for the children’s food packaging design schemes.

### 3.3. CRITIC Indicator Weighting Model and Analysis Process

#### 3.3.1. Data Standardization Processing

Before establishing the indicator weights, the original data must be standardized. Formulas (1) and (2) are used for the standardization of positive and negative indicators, respectively [43].

(1) $$h_{ij}^{*}=\frac{h_{ij}-h_{min}}{h_{max}-h_{j}}$$

In Formula (1), $h_{ij}$ is the evaluation value of the i-th design scheme under the j-th attribute in the original decision matrix. $h_{j}$ is the average of all evaluation values for the j attribute across all design schemes. $h_{max}$ is the maximum value of all positive indicators. $h_{min}$ is the minimum value of all positive indicators. $h_{ij}^{*}$ is the standardized value of the i-th design scheme under the j-th positive indicator.

(2) $$h_{ij}^{-}=\frac{h_{max}-h_{ij}}{h_{max}-h_{j}}$$

In Formula (2), the symbols are the same as those in the formula for positive indicators, but their meanings are reversed. $h_{ij}^{-}$ represents the standardized value of the i-th design scheme under the j-th negative indicator.

In practical application, it is first necessary to determine whether each indicator is positive or negative, and then apply the corresponding formula for standardization. This approach provides a clearer view of how each design scheme performs across various evaluation indicators and where they stand in relation to the optimal (or worst) scheme.

#### 3.3.2. Determination of Evaluation Indicator Weights

The CRITIC model aims to calculate the contrast, conflict, and information entropy of the indicators, thereby scientifically allocating the weights for the 11 sustainability indicators. This provides the core basis for the subsequent use of the MABAC model to rank the sustainability performance of the 20 children’s food packaging designs and guide design optimization. The steps are as follows:

Step 1: Establish the contrast of the indicators.

The contrast is determined by calculating the contrast intensity of each indicator in relation to all other indicators.

The formula for calculating the contrast Formula (3) is as follows:

(3) $$C_{i}=\sqrt{\sum_{i=1}^{m}\left(h_{ij}^{*}-\bar{h_{j}^{*}}\right)/(m-1)}$$

In Formula (3), $C_{i}$ represents the contrast of the j-th indicator, $\bar{h_{j}^{*}}$ represents the average value of all indicators $h_{j}$, and m is the total number of indicators, with m − 1 indicating the total number of indicators minus one.

Step 2: Calculate the indicator conflict.

The formula for calculating the correlation coefficient $R_{ij}$ Formula (4) is as follows:

(4) $$R_{ij}=\frac{\sum_{i=1}^{n}\left(x_{i}-\bar{x_{i}}\right)\left(x_{j}-\bar{x_{j}}\right)}{\sqrt{\sum_{i=1}^{n}{\left(x_{i}-\bar{x_{i}}\right)}^{2}\sum_{i=1}^{n}{\left(x_{j}-\bar{x_{j}}\right)}^{2}}}$$

In Formula (4), $\bar{x_{i}}$ represents the average value of all schemes for indicator $x_{i}$, $\bar{x_{j}}$ represents the average value of all schemes for indicator $x_{j}$, $R_{ij}$ denotes the correlation coefficient between indicator $x_{i}$ and a specific reference indicator.

The formula for calculating conflict $D_{i}$ Formula (5) is as follows:

(5) $$D_{i}=\sum_{i=1}^{m}\left(1-R_{ij}\right)$$

In Formula (5), $D_{i}$ represents the contradiction of the i-th indicator, and ∑ denotes the mathematical symbol for summation, indicating that the following expression should be summed over all relevant j. $1-R_{ij}$ is each term in the summation, where $R_{ij}$ represents the similarity coefficient between the i-th and j-th indicators.

Step 3: Calculate the information content.

In the CRITIC model, the information content represents the richness of information provided by each evaluation indicator during the decision-making process. The formula for calculating information content Formula (6) is as follows:

(6) $$S_{i}=\frac{1}{n-1}\sum_{j=1,j\neq i}^{n}\frac{{\left(z_{i}-z_{j}\right)}^{2}}{\sum_{k=1}^{n}{\left(z_{K}-\bar{z}\right)}^{2}}$$

Here, $S_{i}$ represents the information content of the i-th indicator, n is the total number of evaluation indicators, $z_{i}$ and $z_{j}$ are the standardized values of the i-th and j-th indicators, respectively, and $\bar{z}$ is the average of the standardized values of all indicators.

Step 4: Determine the indicator weights.

The comprehensive allocation of weights involves combining the results from the calculations of contrast, conflict, and information content to determine the weight of each evaluation indicator.

The formula for calculating the weight $W_{i}$ (Formula (7)) is as follows [44]:

(7) $$W_{i}=\frac{C_{i}\times \left(D_{i}\right)}{S_{i}}$$

In the formula, $W_{i}$ represents the weight of the i-th indicator, $C_{i}$ is the contrast, $D_{i}$ is the contradiction, and $S_{i}$ is the information content.

This formula measures the relative importance of indicators through contrast while adjusting the weights based on conflict to reflect the inconsistencies between indicators.

### 3.4. MABAC Multi-Attribute Boundary Approximation Model and Analysis Process

#### 3.4.1. Standardized Decision Matrix

The formula for the weighted standardized decision matrix $R_{ij}$ (Formula (8)) is as follows:

(8) $$O_{ij}=W_{j}\times Z_{ij}$$

In the formula, $O_{ij}$ is an element of the weighted standardized decision matrix, representing the weighted value of the i-th scheme on the j-th indicator. $W_{j}$ is the weight of the j-th indicator, reflecting its importance in the overall evaluation. $Z_{ij}$ is the standardized value of the i-th scheme on the j-th indicator.

#### 3.4.2. Boundary Approximation Region Matrix

Construct the Boundary Approximation Region (BAR) matrix, which includes the positive ideal solution and the negative ideal solution. These two solutions represent the best and worst scenarios, respectively, across all evaluation indicators. The positive ideal solution refers to the maximum weighted value achieved for all positive indicators, while the negative ideal solution refers to the minimum weighted value for the positive indicators.

The construction of the BAR matrix can be achieved using Formula (9):

(9) BAR=maxRijif j is a benefit criterionminRijif j is a cost criterion

In the formula, $max\left(R_{ij}\right)$ represents the maximum weighted value of the j-th indicator among all positive indicators, and $min\left(R_{ij}\right)$ represents the minimum weighted value of the j-th indicator among all negative indicators.

#### 3.4.3. Boundary Approximation Region Distance

The formula for calculating the distance of each sample to the Boundary Approximation Region matrix Formula (10) is as follows:

(10) $$D_{i}=\sqrt{\sum_{j=1}^{n}{\left(R_{ij}-{\bar{R}}_{j}\right)}^{2}}$$

In this formula, $D_{i}$ represents the distance of the i-th scheme to the boundary approximation region. $R_{ij}$ is the weighted value of the i-th scheme on the j-th indicator. ${\bar{R}}_{j}$ is the value of the j-th indicator in the boundary approximation region, representing the ideal or worst performance for that indicator. The distance calculation takes into account the performance of the scheme on each indicator, comparing it to the ideal or worst-case scenario.

#### 3.4.4. Total Distance to the Boundary Approximation Region

The total accumulated distance takes into account the overall performance of each scheme across all evaluation indicators, providing a comprehensive assessment of how closely each scheme approaches the ideal solution.

This process is calculated using Formula (11) [45]:

(11) $${TD}_{i}=\sum_{j=1}^{n}D_{ij}$$

In the formula, ${TD}_{i}$ represents the total distance of the i-th scheme to the boundary approximation region. $D_{ij}$ is the distance of the i-th scheme on the j-th indicator to the boundary approximation region, and n is the total number of evaluation indicators.

Calculating the total distance involves identifying the positive indicators (where higher values are better) and the negative indicators (where lower values are better). The distance of each scheme from the ideal and negative ideal solutions for these indicators is computed and summed to derive the total distance, and finally, the schemes are ranked accordingly. It is important to note that schemes with smaller total distances are ranked higher.

### 3.5. The Optimized Design Method for Children’s Food Packaging

Based on consumer demands and the quantitative results, the study selected the best-performing sustainability scheme, S1, as the reference design for optimizing children’s food packaging. The research further focused on the design of packaging for children’s fruit puree as a practical case for optimization. B. Bossink, from an ecological perspective, pointed out that consumers’ environmental awareness and their attitudes toward eco-friendly packaging significantly influence their purchasing decisions. A positive attitude toward eco-friendly packaging can predict their willingness to pay a higher price for food products [46]. Therefore, the sustainable design optimization of children’s food packaging schemes was carried out from the perspective of R1–R11 indicators.

First, for Environmentally Friendly Materials (R1), the design scheme selected fully biodegradable plastics. The main differences between biodegradable plastics and conventional plastics lie in their material composition, production standards, and decomposition time. Biobased plastics, such as PLA, PHAs, PBA, and PBS, exhibit excellent biodegradability, decomposing into carbon dioxide and water under certain conditions, with the ability to degrade quickly, thereby reducing long-term environmental impacts [47]. Polylactic acid (PLA), a compostable thermoplastic made from renewable resources, is known for its antimicrobial and antioxidant properties, making it suitable for food packaging [48]. In contrast, conventional plastics like PE (polyethylene) are non-biodegradable and take much longer to decompose. Despite the environmental benefits of biobased plastics over conventional plastics, there are no visible differences between them in appearance, making it difficult for consumers to distinguish between the two without product labels and information [49].

Second, for Minimalist and Reduced Design (R2), the packaging design should avoid excessive decoration, using simple patterns and colors to reduce visual clutter and highlight the product itself. The packaging material is minimized by optimizing the structure to reduce material consumption, while ensuring that the protective function of the packaging remains intact. Research by Haiying Wang et al. suggests that images have the most significant influence on consumer purchasing decisions, emphasizing the importance of visual elements in food packaging design [50]. Illustrations representing the fruit puree flavors are carefully selected and designed for the packaging, with strict control over the proportion of illustrations to enhance the minimalist design [51]. Additionally, reducing the number of packaging layers and utilizing a compact layout helps achieve material reduction. These comprehensive measures enable the packaging to meet environmental and sustainability goals without sacrificing functionality or appeal.

Third, for the Lifecycle Environmental Impact Assessment (R3), Z. Boz et al. pointed out that consumer preference for biobased cellulose materials and label claims, along with pre-evaluation education, significantly influence their choices [52]. The use of fully biodegradable plastics reduces dependency on non-renewable resources. Camilleri T. advocates for eco-friendly inks, recommending the use of environmentally friendly inks and UV-curable inks instead of traditional solvent-based inks, to further reduce volatile organic compound (VOC) emissions, thus minimizing the environmental impact [53]. Additionally, simplifying the packaging structure by reducing the number of layers without compromising functionality and protection is encouraged.

Fourth, from a Safety perspective, attention is focused on Material Safety (R4). Morgana Macena et al. found that most consumers consider food safety and quality when purchasing products [54]. This design uses BPA-free, food-grade materials to ensure no harmful substances are present, protecting children’s health. The fruit puree ingredients must meet food safety standards and have good sealing and stability to maintain freshness and prevent contamination. C.S. Costa et al. highlighted that front-of-package nutrition labeling models make it easier for consumers to understand the health aspects of a product, helping make excess nutrients visible and informative [55]. Therefore, this design ensures transparency in material composition, allowing consumers to clearly understand the sources and components of the packaging materials, enhancing the safety and environmental friendliness of children’s fruit puree packaging.

Fifth, for Child Protection Design (R5), Guoqiang An mentioned that safety is the primary consideration in children’s food packaging design. The choice of materials, structural design, and ease of opening are critical to ensuring child protection [56]. The scheme incorporates an easy-open, leak-proof structure, avoiding difficult-to-operate sealing methods, and minimizing the risk of injury during use. The packaging size is ergonomically designed to fit a child’s hand for easy handling and control. Additionally, clear labeling is provided, offering necessary product information and safety warnings for parents to quickly identify and guide children in the proper use of the product.

Sixth, to maximize Ease of Use and Convenience (R6), children, as a special consumer group, have different psychological and physiological characteristics compared to adults. It is essential to consider children’s usage habits and safety to minimize accidents that could occur without adult supervision [57]. This packaging design incorporates a knob-style cap that allows users to open it with one hand, making the process simple and intuitive, without requiring complex skills or tools, thus reducing the risk of injury. To further enhance convenience, the packaging is designed in a flat shape, saving space, and making it easier to stack and store.

Seventh, regarding Freshness and Protective Performance (R7), the scheme uses a high-barrier material, such as an aluminum foil layer, which effectively blocks oxygen and moisture, thereby extending the shelf life of the fruit puree and maintaining its freshness. Advanced sealing technology is employed to ensure that the packaging remains completely sealed until it is opened. Key information such as storage conditions, production date, and expiration date is clearly marked on the packaging to help consumers understand the product’s freshness and safe usage timeframe [53]. Additionally, considering that fruit puree may require multiple uses after opening, the knob-style cap design allows for resealable packaging, reducing the exposure of food to air and maintaining its quality and taste.

Eighth, in terms of Environmental Education Elements (R8) and Interactive Educational Function (R9), Norton V and Alexi N pointed out in their research that children’s packaging often lacks clear and understandable information about proper packaging disposal, and children need to learn sustainable behaviors from an early age [14]. Therefore, the design includes environmental-themed stories and small puzzles printed on the packaging to spark children’s curiosity and encourage them to explore and learn. Parents can use these stories and puzzles to interact with their children, discussing the environmental actions depicted in the stories and solving the puzzles together, thus enhancing parent–child communication. Khloud Ahmed suggests the use of technologies like QR codes, linking the packaging to online educational resources that provide additional learning materials and interactive experiences, as well as delivering more environmental information [41]. Children can scan the QR code on the packaging with a smartphone or tablet to access an interactive education platform where they can learn about environmental knowledge through fun activities.

Ninth, regarding Cost-Effectiveness Analysis (R10), the scheme evaluates the cost of different environmentally friendly materials, including recyclable or biodegradable materials, comparing them with the currently used fully biodegradable plastic. P. Bałdowska emphasizes the need to assess the environmental impact of packaging materials, including the source of the materials, energy consumption during production, and emissions. This involves conducting a lifecycle analysis of the material, considering environmental impacts at all stages from raw material extraction to final product manufacturing [58]. The scheme calculates the cost coefficients at each stage of packaging production. Market research is conducted to understand target consumers’ acceptance of and willingness to pay for eco-friendly packaging, as well as to analyze competitors’ pricing strategies and market performance to determine the product’s price point and market positioning.

Tenth, in enhancing Market Competitiveness (R11), the scheme first optimizes the design by understanding the expectations and needs of parents and children regarding eco-friendly packaging, ensuring that the design aligns with their values and usage habits. Innovative eco-friendly materials are used to ensure that packaging safety and functionality are not compromised, meeting parents’ primary concerns about food safety for their children [59]. Additionally, the design incorporates bright colors such as pink, purple, and orange, as well as fun patterns and educational content to capture children’s attention while conveying positive environmental messages. Storytelling elements and interactive features, such as QR codes linked to educational content, enhance the educational and entertainment value of the packaging. Lastly, the scheme focuses on brand building and market promotion through social media, public relations, and brand partnerships to raise brand awareness and improve its positive image. Continuous monitoring of market feedback and consumer behavior enables the company to respond quickly to market changes, ensuring ongoing optimization of product design and marketing strategies. In summary, the method for converting the sustainability evaluation indicators into design elements is shown in Table 4.

## 4. Results

### 4.1. Characteristics of the Consumer Sample Group

Surveying parent consumer groups in the design of children’s food packaging is crucial, as parents are the key influencers in purchasing decisions for children’s food. Research by C. Elliott et al. indicates that the packaging of children’s food significantly affects children’s product preferences and parental purchasing decisions [35]. By analyzing the sample, we gain deeper insights into parents’ expectations and preferences regarding the sustainability evaluation indicators of children’s food packaging. The consumer sample exhibits a balanced and representative distribution across multiple dimensions, such as gender, age, educational background, professional field, and income level. This diverse sample enriches the study’s depth and breadth, uncovers specific needs across different consumer groups in children’s food packaging design.

As seen from the data in Table 3, 60% of the sample group consists of female consumers, while males account for 40%, showing a relatively balanced gender ratio. This indicates that the study covers the consumption views and preferences of parents from both genders, ensuring gender representativeness in the results. The age structure reveals that the 26–31 age group has the highest proportion of parents, at 40%, followed by the 32–37 age group, accounting for 28%. These two age groups together make up 68% of the sample, indicating that middle-aged parents are the primary demographic focused on children’s food packaging. Parents in the 20–25 age group account for 20%, while those in the 38–43 age group represent 12%, contributing diverse age perspectives to the study despite being smaller portions of the sample.

In terms of education level, 48% of parents have an associate’s degree or university-level education, followed by 40% with a high school education, and 4% with a master’s degree or higher. The relatively high education levels influence their understanding and evaluation of children’s food packaging. Professionally, 32% of the sample are corporate employees, 20% are freelancers, and 24% are government or public sector employees. This distribution indicates that corporate employees form a significant portion of the sample and may have heightened concerns about children’s food packaging. Regarding household monthly income, 48% of families fall within the CNY 2000–5000 income range, followed by 12% in the below CNY 2000 range. This suggests that middle-to-low-income families represent a significant proportion of the sample, and their purchasing power and preferences have an important impact on the children’s food packaging market.

### 4.2. Evaluation Results of Children’s Food Packaging Design Cases

Based on the scoring data from 250 parents in the consumer group, who evaluated 20 different categories of children’s food packaging schemes (S1–S20) currently available on the market using the 11 sustainability evaluation indicators (R1–R11), each indicator’s score ranges from 1 to 9. This reflects the consumers’ satisfaction with each scheme in these aspects and results in a sustainability scoring table for children’s food packaging design schemes (Table 5). The data in Table 5 provides a quantitative perspective to assess and compare the sustainability performance of different children’s food packaging schemes. For example, Scheme S1 scored higher in Child Protection Design (R5) and Environmental Education Elements (R8), indicating that consumers believe it performs well in protecting children and providing environmental education. On the other hand, Scheme S4 scored higher in Material Safety (R4), suggesting that consumers feel its materials are safer for children. Conversely, if a particular scheme scores low on certain indicators, these areas may require further optimization to improve its sustainability.

Table 6 presents the standardized values of positive indicators for the 20 children’s food packaging design schemes (S1–S20) in the sustainability evaluation. These values were obtained by applying a specific standardization formula (Formula (1)) to the raw scoring data, ensuring fair comparison across different indicators. The purpose of the standardization process is to convert the original scores into values within a range of 0 to 1, where 1 represents the ideal state for that indicator and 0 represents the least ideal state. By standardizing the scores, it becomes easier to directly compare different design schemes, regardless of the original scale or units used for each indicator. According to this status indicator, the performance of different design schemes across various sustainability indicators can be compared more intuitively, as the standardized values offer a clear and consistent way to assess how close each scheme is to the optimal performance.

By applying Formula (2), the standardized values of negative indicators for the 20 children’s food packaging design schemes in the sustainability evaluation are obtained (Table 7). Unlike positive indicators, negative indicators are characterized by lower values being better. From the data in Table 7, it is evident that the performance of different design schemes varies across negative indicators. Some design schemes score close to 0 on certain negative indicators, indicating that their performance in these specific areas is near the ideal state. On the other hand, other design schemes may score closer to 1 on the same indicators, signifying less favorable performance in those aspects. For example, Scheme S1 has a score of 0.00 on the Material Safety (R4) indicator, indicating that its material safety is very high, approaching the ideal state. In contrast, Scheme S2 has a score of 0.50 on the same indicator, suggesting there is still room for improvement in this aspect.

### 4.3. Indicator Weight Values

To calculate the conflict between indicators, it is first necessary to compute the correlation coefficients of the sustainability evaluation indicators using Formula (4) (Table 8). The correlation coefficient ranges from −1 to 1, where 1 indicates a perfect positive correlation, −1 indicates a perfect negative correlation, and 0 indicates no linear correlation. From the data in Table 8, it can be seen that the correlation between different indicators varies. Some indicators have a strong positive correlation, such as the correlation coefficient between Ease of Use and Convenience (R6) and Freshness and Protective Performance (R7), which is 1.00. This indicates that these two indicators tend to change in tandem across the design schemes. On the contrary, there are negative correlations between certain indicators, such as Cost-Effectiveness (R10) and Market Competitiveness (R11), with a correlation coefficient of −0.67. This suggests that an improvement in cost-effectiveness might reduce market competitiveness, or vice versa. Additionally, some indicators have a weak or no significant linear relationship with others, such as the correlation coefficient between Environmentally Friendly Material Selection (R1) and Minimalist Design (R2), which is 0.50. This shows that while there is some association between these two indicators, the relationship is not particularly strong.

The contrast of the sustainability evaluation indicators for children’s food packaging design was derived using Formula (3), and the conflict between indicators was calculated using Formula (5). The information content weight of each evaluation indicator was quantified using Formula (6). Finally, through Formula (7), the comprehensive weights of the sustainability evaluation indicators for children’s food packaging design were obtained, as shown in Table 9. Contrast measures how well an indicator differentiates between different design schemes, with higher contrast indicating that the indicator can more effectively distinguish between schemes. Conflict reflects the inconsistency between an indicator and other indicators, where higher conflict might suggest that this indicator may be in opposition to other sustainability goals. Information content refers to the importance of each indicator in the overall decision-making process, while the weight is a combined result of contrast, conflict, and information content, reflecting the relative importance of each indicator in the overall evaluation system. From the data in Table 9, it is clear that different indicators perform differently in these attributes. For example, R4 (Material Safety) shows high contrast and weight, indicating that it plays an important role in distinguishing different design schemes and in the overall evaluation. On the other hand, R6 (Ease of Use and Convenience) has a lower weight, suggesting that its relative importance in the sustainability evaluation is lower. The results of the study highlight that among all the sustainability evaluation indicators, Environmentally Friendly Material Selection (R1), Material Safety (R4), and Freshness and Protective Performance (R7) carry the most significant weights. This suggests that these indicators should be given more consideration in the sustainability assessment of children’s food packaging design.

### 4.4. Sustainability Ranking

Step 1: Calculate the Weighted Standardized Decision Matrix.

Based on Formula (8), the study quantified the performance of each children’s food packaging design scheme across different dimensions by combining the weight of each evaluation indicator with the sustainability scores of the schemes. By assigning corresponding weights to each indicator, the weighted decision matrix was constructed as shown in Formula (12). The weighted decision matrix provides an intuitive comparison tool, enhancing the focus and accuracy of the evaluation process.

(12) $$O_{ij}=\left[\begin{array}{c} \begin{array}{c} 0.128,\;\;\;0.138,\;0.115,\;\;0.115,\;\;0.154,\;\;0.115,\;\;0.138,\;\;0.115,\;\;0.096,\;\;0.107,\;\;0.132,\\ 0.115,\;\;0.123,\;\;0.096,\;0.096,\;\;0.134,\;\;0.096,\;\;0.123,\;\;0.115,\;\;0.077,\;\;0.123,\;\;0.121, \end{array}\\ 0.141,\;\;0.107,\;\;\;0.134,\;0.115,\;0.115,\;\;0.134,\;\;0.107,\;\;0.134,\;\;0.154,\;\;0.107,\;\;0.099,\\ 0.102,\;\;0.154,\;\;0.077,\;\;0.134,\;\;0.096,\;\;0.077,\;\;0.154,\;\;0.154,\;\;0.115,\;\;0.092,\;0.143,\\ 0.128,\;\;0.092,\;\;0.154,\;\;0.154,\;\;0.134,\;\;0.154,\;\;0.092,\;\;0.077,\;\;0.134,\;\;0.107,\;0.121,\\ 0.115,\;\;0.138,0.134,\;0.115,\;\;0.115,\;\;0.096,\;\;\;0.138,\;\;0.096,\;\;0.096,\;\;0.123,\;\;0.154,\\ 0.141,\;0.123,\;\;0.115,\;0.096,\;\;0.077,\;\;0.134,\;\;0.123,\;\;\;0.115,\;0.115,\;\;\;0.138,\;\;0.132,\\ 0.154,\;\;0.107,\;0.096,\;\;0.134,\;\;0.115,\;\;0.134,\;\;0.123,\;\;0.134,\;\;0.077,\;\;0.092,\;\;0.121,\\ 0.102,\;\;0.138,\;\;\;0.134,\;\;0.134,\;\;0.096,\;\;0.115,\;\;0.107,\;\;0.115,0.154,\;0.123,\;\;0.110,\\ 0.128,\;\;0.092,\;\;\;0.115,\;0.096,\;\;0.134,\;0.154,\;\;0.092,\;\;0.154,\;\;0.115,\;\;0.138,\;\;0.088,\\ 0.115,\;\;0.123,\;\;0.096,\;\;0.154,\;\;0.134,\;\;0.096,\;0.138,\;\;0.115,\;\;0.134,\;\;0.077,\;\;0.143,\\ 0.141,\;\;0.107,\;\;0.134,\;0.115,\;\;0.115,0.134,\;\;0.107,\;\;0.134,\;\;0.096,\;\;0.154,\;\;\;0.099,\\ 0.102,\;\;0.154,\;\;0.077,\;\;0.134,\;\;0.096,\;0.077,\;\;0.154,\;\;0.154,\;\;0.115,\;\;0.092,\;\;0.132,\\ 0.128,\;\;0.092,\;\;0.154,\;\;0.154,\;\;0.077,\;\;0.154,\;\;0.092,\;\;0.077,\;\;0.134,\;\;0.107,0.099,\\ 0.115,\;0.138,0.134,\;\;\;0.115,\;\;0.115,\;\;0.096,\;\;0.138,\;\;0.096,\;\;0.096,\;\;0.123,\;\;0.154,\\ 0.141,\;\;\;0.123,\;\;0.115,\;\;0.096,\;0.077,\;\;0.134,\;\;0.123,\;\;0.134,\;0.115,\;\;0.138,\;\;0.132,\\ 0.154,\;\;0.107,\;\;0.096,\;\;0.134,\;\;0.115,\;0.134,\;\;0.123,\;\;0.134,\;\;0.077,\;\;0.092,\;0.121,\\ 0.102,\;\;0.138,\;\;0.134,\;\;0.134,\;0.096,\;\;0.115,\;\;0.107,\;\;0.115,\;\;0.154,\;\;0.123,\;\;0.110,\\ 0.071,\;\;0.077,0.096,\;\;0.077,\;\;0.115,\;\;0.134,\;\;0.077,\;\;0.134,\;\;0.096,\;0.123,\;\;0.077,\;\\ 0.115,\;\;\;0.123,0.096,0.154,\;\;0.134,\;\;0.096,\;\;0.138,\;\;0.115,\;\;0.134,\;\;0.077,\;\;0.143 \end{array}\right]$$

Step 2: Calculate the Boundary Approximation Region Matrix.

By quantifying the boundary conditions for scheme evaluation, a comprehensive evaluation of the sustainability of children’s food packaging design schemes can be conducted under a unified quantitative standard. Using Formula (9), the Boundary Approximation Region Matrix is derived as shown in Formula (13).

(13) $$BAR=\left[0.108,0.105,0.1010,0.108,0.099,0.104,0.1056,0.107,0.099,0.099,0.107\right]$$

Step 3: Calculate the Distance Between the Schemes and the Boundary Approximation Region.

Through mathematical modeling, qualitative analysis is transformed into quantitative evaluation, enhancing the accuracy and reliability of the decision-making process. This allows for an objective comparison of the sample schemes. Using Formula (10), the distances between the schemes and the boundary approximation region are calculated as shown in Formula (14).

(14) $$D_{i}=\left[\begin{array}{c} \begin{array}{c} 0.020,\;0.033,\;0.014,\;\;0.007,\;\;0.056,\;\;0.011,\;\;0.033,\;\;0.009,-0.003,\;0.009,\;0.025\\ 0.007,\;0.017,-0.005,-0.012,\;0.036,-0.008,\;0.018,\;0.009,-0.02,\;0.024,\;0.014, \end{array}\\ 0.032,\;\;0.002,\;\;0.033,\;0.007,\;0.017,\;\;0.030,\;0.002,\;0.028,\;0.054,\;0.009,-0.008,\\ -0.005,\;0.048,-0.024,\;0.026,-0.002,-0.027,\;0.05,\;0.05,\;0.02,-0.007\;0.04\\ 0.02,-0.013,\;0.053,\;0.046,\;0.036,\;0.050,-0.013,-0.030,\;0.035,\;0.009,\;0.014,\\ 0.007,\;0.033,\;0.034,\;0.007,\;0.017,-0.008,\;0.033,-0.010,-0.003,\;0.024,\;0.04,\\ 0.032,\;\;0.017,\;0.015,-0.012,-0.021,\;0.030,\;0.018,\;0.009,\;0.01,\;0.040,\;0.025,\\ 0.046,\;0.002,-0.005,\;0.026,\;0.017,\;0.030,\;0.018,\;0.03,-0.023,-0.007,\;0.014,\\ -0.005,\;\;0.033,\;0.034,\;0.026,-0.002,\;0.011,\;0.002,\;0.009,\;0.054,\;0.024,\;0.003,\\ 0.02,-0.013,\;0.015,-0.012,\;0.04,\;0.05,-0.013\;,0.048,\;0.016,\;0.04,-0.019,\\ 0.007,\;0.018,-0.005,\;0.046,\;0.04,-0.008,\;0.03,\;0.009,\;0.035,-0.022,\;0.036,\\ 0.033,\;0.002,\;0.034,\;0.007,\;0.017,\;0.03,\;0.002,\;0.028,-0.003\;,0.055,-0.008,\\ -0.005,\;0.048,-0.02,\;0.02,-0.002,-0.03,\;0.05,\;0.048,\;0.016,-0.007,\;0.025,\\ 0.02,-0.01,\;0.05,\;0.046,-0.021,\;0.05,-0.013,-0.03,\;0.035,\;0.009,-0.008,\\ 0.007,\;0.033,\;0.034,\;0.007,\;0.017,-0.008,\;0.03,-0.01,-0.003,\;0.024,\;0.047,\\ 0.032,\;0.017,\;0.014,-0.012,-0.021,\;0.030,\;0.018,\;0.028,\;0.016,\;0.040,\;0.025,\\ 0.04,\;0.002,-0.005,\;0.026,\;0.017,\;0.03,\;0.018,\;0.028,-0.023,\;-0.007,\;0.014,\\ -0.005,\;0.033,\;\;0.033,\;\;0.026,\;-0.002,\;0.011,\;0.002,\;0.009,\;0.054,\;0.024,\;0.003,\\ -0.03,-0.02,-0.005,-0.03,\;0.01,\;0.03,-0.02,\;0.02,-0.003,\;0.02,-0.03,\\ 0.007,\;\;0.017,\;-0.005,\;0.04,\;0.03,-0.008,\;\;0.03,\;\;0.009,\;0.03,-0.02,\;0.0360 \end{array}\right]$$

The total distance to the Boundary Approximation Region provides a clear basis for ranking the sustainability of the 20 children’s food packaging design samples. Using Formula (11), the total distance to the Boundary Approximation Region is calculated, and the schemes are ranked in descending order based on the total distance. The ranking results are shown in Table 10.

The specific ranking results are as follows: S1 > S3 > S5 > S12 > S9 = S18 > S16 > S11 = S20 > S6 = S15 > S7 > S10 > S4 > S8 = S17 > S13 > S14 > S2 > S19.

### 4.5. The Results of the Optimized Design Scheme for Children’s Fruit Puree Packaging

**Figure 4:**
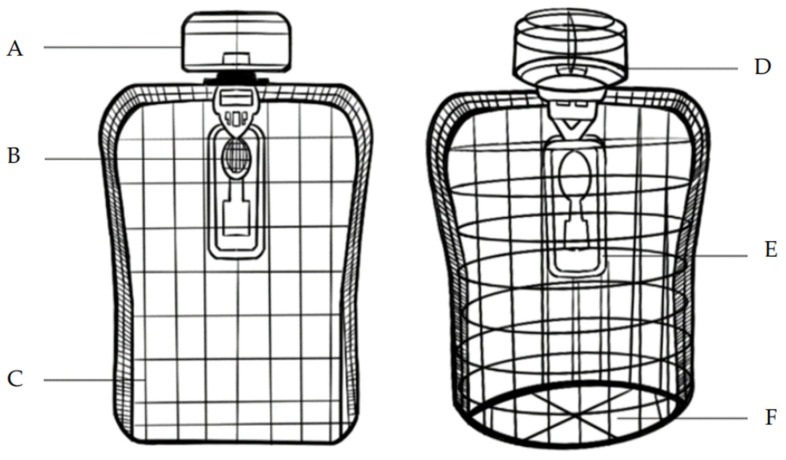
Structural diagram of children’s fruit puree packaging.

Based on the various indicator systems outlined in Table 10’s design layer, the study further developed the optimized design scheme for children’s fruit puree packaging. The research first optimized the structural details of the case design, integrating the sustainable design indicator system and evaluation standards. The resulting optimized scheme for children’s fruit puree packaging design is illustrated in Figure 4.

In this design scheme:

A: Represents the design of the fruit puree packaging cap and toy car wheels, combining packaging reusability with children’s educational development. By extending the packaging’s lifecycle, it stimulates children’s creativity and hands-on abilities. The integration of environmental concepts with children’s play allows them to learn about resource recycling while having fun.

B: Refers to the ergonomic silicone spoon designed for the fruit puree, enhancing convenience and safety during consumption. The soft edges of the silicone spoon prevent children from injuring themselves while eating, ensuring comfort and safety during use.

C: Indicates the use of fully biodegradable plastic for the fruit puree packaging pouch. This material can naturally degrade after use, reducing its environmental impact.

D: Represents the sealing technology at the cap, which strengthens the packaging’s seal. This ensures that the fruit puree remains fresh before opening, extends its shelf life, and prevents spoilage.

E: Refers to the design of the transparent storage case for the silicone spoon, focusing on the product’s added value and post-use convenience. The silicone spoon comes with a transparent case that not only provides dust protection but also allows for easy storage after use, keeping the spoon clean and ready for repeated use.

F: Refers to the round-bottom design of the fruit puree packaging, mainly considering the practicality and stability of the packaging, making it easy to place and use.

**Figure 5:**
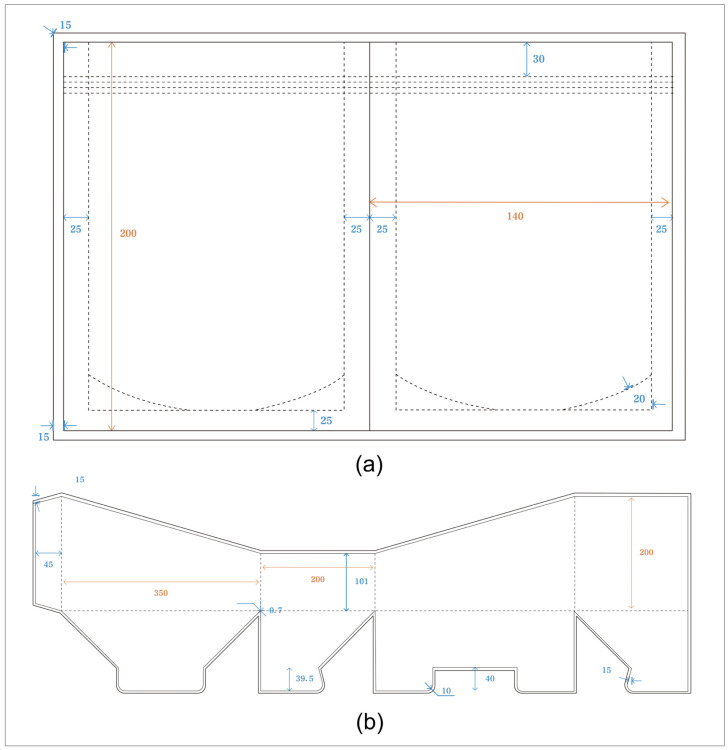
The unfolded diagram of the fruit puree packaging pouch and display box (unit: mm). (**a**) Unfolded diagram of the fruit puree packaging pouch; (**b**) Unfolded diagram of the slanted display box.

The die-cut and scale diagram of the fruit puree packaging pouch and display box are shown in Figure 5.

**Figure 6:**
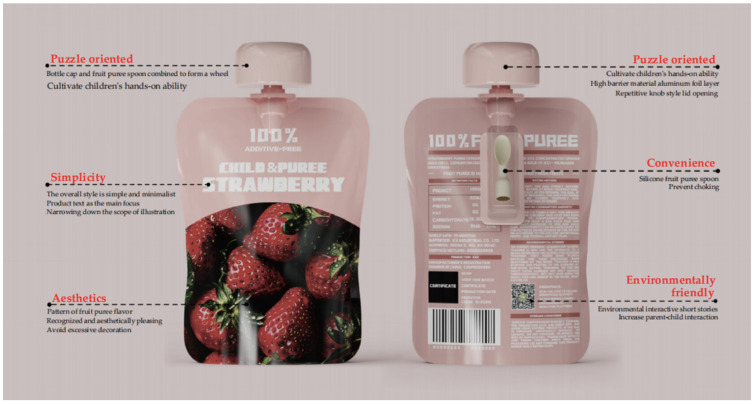
Front and back detail images of the fruit puree packaging sample.

In the overall design of the children’s fruit puree packaging, the scheme is simple and clear, with illustrations representing the puree flavors as the focal point to attract users’ attention. The label “100% Pure Fruit Puree” is prominently displayed, helping consumers recognize the product’s natural qualities. The packaging uses high-barrier aluminum foil combined with cap sealing technology to enhance freshness protection, effectively extending the shelf life of the puree and ensuring its freshness. Important information such as storage conditions, production date, and expiration date is clearly indicated on the packaging, ensuring that consumers can accurately understand the product’s freshness and safe consumption period. Additionally, the packaging features an environmental story and a QR code that links to an environmental education platform, allowing children and parents to learn about sustainability together, which enhances parent–child interaction, as shown in Figure 6.

**Figure 7:**
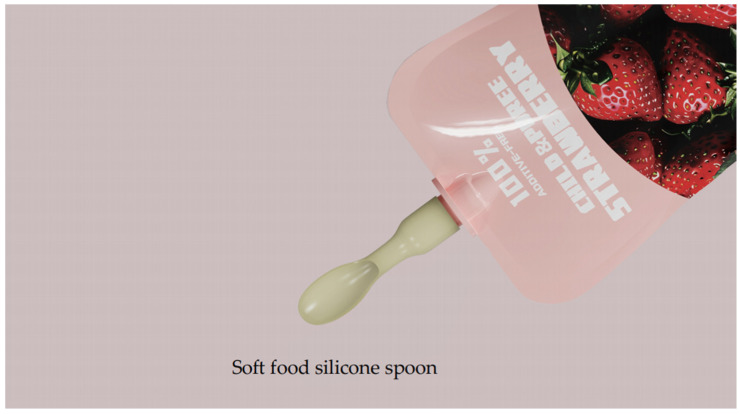
Detailed usage images of the silicone fruit puree spoon.

The fruit puree spoon included with the packaging is made of silicone material, which is soft and safe, preventing infants from accidentally choking while eating. It also allows parents to squeeze the puree onto the spoon, controlling the amount for more convenient feeding of infants. As shown in Figure 7.

**Figure 8:**
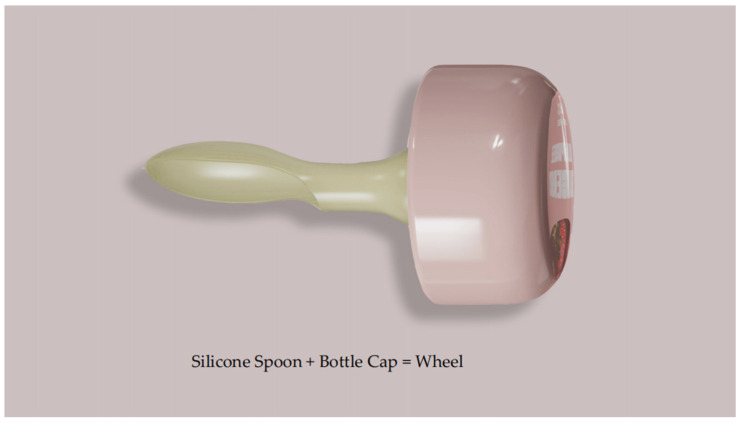
Detailed combination image of the cap and fruit puree spoon.

**Figure 9:**
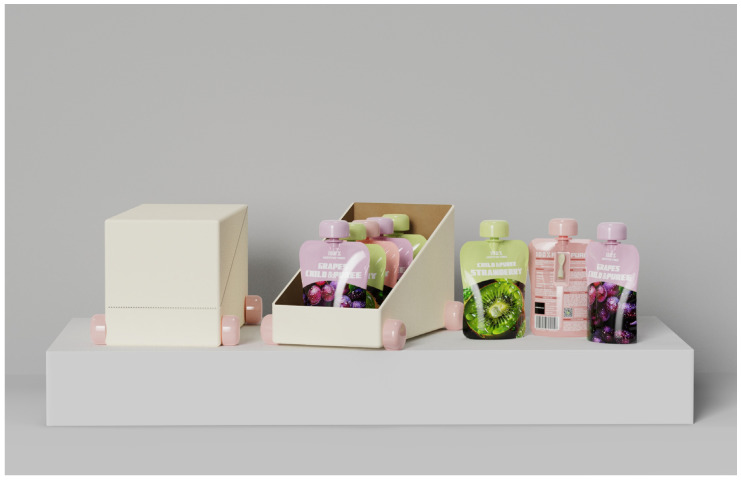
Display image of the assembled children’s fruit puree packaging.

Considering the primary user group of the packaging is children, a knob-style cap design is used, allowing the entire opening process to be completed without the need for any additional tools. This ensures that children can safely and independently open the package. The cap design also incorporates an educational element—after the fruit puree is consumed, children can remove the cap and combine it with the puree spoon to create wheels for a toy car (Figure 8). Additionally, the cap can be further combined with the display box to create a fun toy car. The display box set not only facilitates in-store presentation, highlighting the playful design, but it can also be repurposed as a storage box at home, maximizing the sustainable reuse of the packaging. As shown in Figure 9.

## 5. Discussion

### 5.1. Quantitative Analysis and Discussion of Indicator Weights

As shown in Table 9, the “Material Safety Indicator” (0.3292236) has the highest contrast among all indicators, followed by the “Lifecycle Environmental Impact Indicator” (0.3034885), ranking second. These two indicators play a decisive role in differentiating various design schemes due to their significant differences. The “Child Protection Design Indicator”, “Environmental Education Elements Indicator”, and “Minimalist Design Indicator” all have the same contrast value (0.2798496), showing equal differentiation across different design schemes. The values for the “Market Competitiveness Indicator” (0.2799225), “Cost-Effectiveness Indicator” (0.2773939), and “Ease of Use and Convenience Indicator” (0.2563521) suggest that while these indicators are not the most prominent in distinguishing between schemes, they are still critical aspects that should not be overlooked in the design process. Using Formula (3), the “Lifecycle Environmental Impact” and “Material Safety” indicators, with the highest contrast values, emerge as key indicators that require attention in different design schemes. Conversely, the “Ease of Use and Convenience” indicator has a relatively lower contrast.

The correlation coefficient matrix (Table 8) reflects the statistical relationships between sustainability indicators in children’s food packaging design, where −1 represents complete negative correlation, 0 represents no correlation, and 1 represents complete positive correlation. The correlation coefficient between “Minimalist and Reduced Design” and “Lifecycle Environmental Impact” is −0.8277, indicating a significant negative correlation, suggesting that minimalist design plays an essential role in reducing the environmental impact throughout the packaging’s lifecycle. The correlation coefficient between “Ease of Use and Convenience” and “Environmental Education Elements” is 0.1973, indicating a positive correlation between these two indicators. This shows that when incorporating environmental education elements in the design, attention should also be given to ease of use, ensuring that one indicator is not prioritized at the expense of the other. The correlation coefficient between “Child Protection Design” and “Environmentally Friendly Material Selection” is 0.0463, which, although low, highlights the complementary relationship between the two indicators. The correlation coefficient between “Cost-Effectiveness Analysis” and “Market Competitiveness Analysis” is −0.374735, indicating a trade-off between these two indicators. An excessive pursuit of cost-effectiveness may negatively affect the product’s market competitiveness. The correlation coefficient between “Ease of Use and Convenience” and “Freshness and Protective Performance” is −0.008742, which is close to 0, indicating that these two indicators are relatively independent and that changes in one do not affect the other.

According to the conflict values in Table 9, indicators such as “Environmental Education Elements” (18.8134424), “Interactive Educational Function” (18.8362331), “Ease of Use and Convenience” (18.6048578), “Market Competitiveness Enhancement” (18.6424832), and “Minimalist and Reduced Design” (18.7328038) have relatively low conflict values. These indicators are relatively easy to coordinate with other objectives. For example, including interactive elements in packaging, such as QR codes linking to environmental knowledge pages, is unlikely to significantly impact the simplicity or cost of the packaging. On the other hand, the “Lifecycle Environmental Impact Indicator” (19.1306695), “Material Safety” (19.1825862), and “Child Protection Design” (19.7801432) exhibit higher conflict values, indicating the need for careful balancing in specific designs. For instance, enhancing child protection may require special packaging mechanisms to prevent children from accidentally opening the package, such as complex seals or locking designs, which could reduce ease of use and convenience, thereby conflicting with the ease-of-use indicator. “Cost-Effectiveness Analysis” (20.1249224), “Freshness and Protective Performance” (20.3670509), and “Environmentally Friendly Material Selection” (20.2434242) have the highest conflict values among all indicators. These indicators face significant conflicts with other goals. For example, strengthening the freshness performance of packaging may require the use of additional protective layers or special preservatives, which could increase costs and environmental burden [60].

The information content of each indicator, calculated using Formula (6) (Table 9), reflects which indicators provide unique perspectives and critical information during the decision-making process. High information content indicators offer rich insights for decision-making across different schemes and should be given more weight in multi-criteria decision-making. This ensures that decision-makers not only focus on contrast and conflict but also balance the richness of information provided by each indicator [61]. “Environmentally Friendly Material Selection” (6.4852165) is the highest among all indicators, highlighting the importance of considering environmentally friendly attributes during material selection. This underscores how preferences for eco-friendly materials can greatly influence sustainability evaluations of packaging. “Material Safety” (6.3153595) ranks second, further emphasizing the need to ensure that packaging materials are safe for children. Indicators such as “Child Protection Design” (5.6210622), “Lifecycle Environmental Impact” (5.8059380), “Cost-Effectiveness Analysis” (5.5825305), “Interactive and Educational Function” (5.5927404), and “Freshness and Protective Performance” (5.9791852) fall within the medium range, indicating that these indicators provide a moderate level of information in the design schemes and should be handled accordingly based on the specific context. “Minimalist Design” (5.24236740), “Environmental Education Elements” (5.2649340), and “Market Competitiveness” (5.2184505) have lower information content, while “Ease of Use and Convenience” (4.7693935) ranks the lowest among all indicators. This suggests that these indicators are relatively less important in the current evaluation framework, and the information they provide is limited in terms of sustainability. However, they should not be entirely disregarded. For instance, while “Market Competitiveness” is critical for businesses, in a sustainability-focused evaluation system, it is not as directly related as “Environmentally Friendly Material Selection.”

Following the steps of the CRITIC method, the contrast of the sustainability indicators was calculated using Formula (3), conflict was evaluated using Formula (5), and information content was determined using Formula (6). Based on these factors, Formula (7) was applied to compute the weight values of each indicator (Table 9). This weight determination not only considers the contrast, conflict, and information richness among the indicators but also reflects their importance in a comprehensive and balanced manner within multi-criteria decision-making [62]. The weight values calculated using the CRITIC method highlight “Environmentally Friendly Material Selection” (0.1048079) as the top indicator, underscoring the importance of material choice in sustainable packaging design. The next highest weighted indicator is “Material Safety” (0.1020628), which emphasizes the direct impact of material safety on children’s health and safety in food packaging design. The indicators “Interactive and Educational Function” (0.0903845), “Child Protection Design” (0.0908423), “Freshness and Protective Performance” (0.0966299), “Cost-Effectiveness Analysis” (0.0902195), and “Lifecycle Environmental Impact” (0.0938300) are given moderate to high weights. This reflects that, in addition to sustainability and safety, the design of children’s food packaging also needs to consider factors such as educational value, child protection, freshness performance, economic benefits, and lifecycle impact, to create packaging that is both appealing to children and responsible in terms of sustainability. The indicators “Market Competitiveness Enhancement” (0.0843356), “Minimalist and Reduced Design” (0.0847221), and “Environmental Education Elements” (0.0850868) are assigned lower weights, while “Ease of Use and Convenience” (0.0770784) holds the lowest weight among all indicators. Although these indicators have relatively lower importance in the sustainability evaluation system, they still play significant roles in the design process. For example, in designing a new type of eco-friendly material packaging, minimalist design can help reduce material usage while enhancing market competitiveness. Additionally, incorporating environmental education elements, such as explaining how to recycle materials on the packaging, is a highlight of sustainable packaging design.

According to the results in Table 8, “Environmentally Friendly Material Selection”, “Material Safety”, and “Freshness and Protective Performance” are the highest-weighted indicators in the sustainability evaluation system. It is worth noting that even if an indicator scores highly in contrast, conflict, or information content, its overall weight may still be relatively small, indicating that its influence on the final result is limited.

### 5.2. Discussion on the Ranking of the Sustainability Performance of S1–S20 Children’s Food Packaging Schemes

The analysis based on the weighted standardized decision matrix Formula (12) constructed using Formula (8) can be summarized as follows:

(a). Horizontal Comparison Between Children’s Food Packaging Sustainability Schemes: For example, S8 scores 0.154 on the “Ease of Use and Convenience” indicator, which is relatively high, while S14 scores 0.154 on the “Environmentally Friendly Material Selection” indicator. This shows that different children’s food packaging sustainability schemes perform differently on specific indicators.

(b). Identifying the Highest and Lowest Scores Among the Sustainability Schemes: By comparing the maximum and minimum values in each column, the value of 0.154 in the “Environmentally Friendly Material Selection” column is the highest, indicating that S5, S10, and S14 perform best in this aspect. This allows us to identify the best and worst-performing schemes on specific indicators.

(c). Considering the Balance of Sustainability in the Packaging Schemes: For instance, S1 has the following scores across the indicators: 0.128, 0.139, 0.116, 0.116, 0.154, 0.116, 0.139, 0.116, 0.096, 0.108, and 0.132. This suggests that S1 performs well across several dimensions, demonstrating a balanced design. Conversely, if a scheme scores very high on some indicators but low on others, it may indicate a lack of balance in the design. For example, S18 scores 0.154 on “Material Safety”, but scores relatively low on other indicators, suggesting that Scheme 18 lacks overall balance.

(d). Understanding the Impact of Weights on Sustainability Schemes: The values in the weighted decision matrix are adjusted according to the assigned weights, meaning certain indicators have a greater influence based on their importance. For instance, S2’s score on the “Material Safety” indicator is only 0.096. Since “Material Safety” carries significant weight, S2 ranks relatively low overall despite its performance on other indicators.

The Boundary Approximation Region Matrix, by defining the boundary between the ideal and negative ideal solutions, provides a more refined decision boundary for evaluation. The ideal solution represents the best performance across all evaluation indicators, while the negative ideal solution represents the worst performance. Based on Formula (9), the Boundary Approximation Region Matrix Formula (13) shows that “Interactive and Educational Function” (0.1084465) ranks first, indicating that this indicator has the best ideal solution among all evaluation criteria. This means that achieving high levels of interaction and educational function in children’s food packaging design is of utmost importance, and packaging schemes with these features may be more favored. “Ease of Use and Convenience” (0.1083439) ranks second, showing that ease of use remains a critical factor for consumers when judging children’s food packaging. The “Material Safety” indicator value (0.1065876) ranks third, emphasizing the importance of material safety in children’s food packaging design. “Child Protection Design” (0.0984984) ranks the lowest, suggesting that improvements are most needed in this area among all the evaluation indicators.

Formula (10) calculates the distance between each scheme and the Boundary Approximation Region, which helps evaluate and compare the gap between each alternative scheme and the ideal or negative ideal solution [63]. As shown in Formula (14), the positive or negative distance values reflect whether the scheme performs better (positive) or worse (negative) than the baseline for a particular indicator. For example, S1 has a distance value of 0.020 for the “Ease of Use and Convenience” indicator, indicating that S1 performs better than the negative ideal solution on this indicator. A negative value, on the other hand, suggests that the scheme performs worse than the ideal solution. For instance, S2 has a distance value of −0.05 for the “Lifecycle Environmental Impact” indicator, meaning S2’s performance on this indicator is below the ideal solution. The magnitude of the distance shows how close the scheme is to the ideal state—a smaller positive value indicates that the scheme is close to the ideal solution, while a larger negative value means the scheme is far from the ideal solution. For example, S6 has a distance value of 0.007 on the same “Ease of Use and Convenience” indicator, which is smaller than S7’s 0.033, indicating that S6 is closer to the ideal solution on this indicator. By comparing the distance values of different schemes on the same indicator, the performance of each scheme can be evaluated. For instance, S18 has a distance value of 0.009 on the “Freshness and Protective Performance” indicator, while S19’s corresponding value is 0.028, indicating that S18 performs closer to the ideal state on this indicator.

Formula (11) calculates the total distance of each scheme to the Boundary Approximation Region (Table 10). According to the total distance, the schemes are ranked from best to worst based on how far they are from the ideal solution—the greater the distance, the better the scheme; the closer the distance, the worse the scheme [63]. The ranking of the 20 children’s food packaging design schemes (S1–S20) based on total distance in descending order is: S1 (0.214) > S3 (0.209) > S5 (0.207) > S12 (0.198) > S9 (0.190) = S18 (0.190) = S1 (0.190) > S11 (0.185) = S20 (0.185) > S6 (0.181) = S15 (0.181) > S7 (0.169) > S10 (0.166) > S4 (0.157) > S8 (0.148) = S17 (0.148) > S13 (0.146) > S14 (0.127) > S2 (0.079) > S19 (−0.058). The ranking results show that S2 has the smallest total distance (0.079), indicating the smallest deviation from the ideal solution across all evaluation indicators, making it the best-performing scheme overall and deserving the highest priority as the preferred design solution. S19 has a negative total distance (−0.058), indicating that it performs worse than the ideal solution on certain indicators.

## 6. Conclusions

The study utilized a questionnaire to collect actual rating data from 250 parent consumers and established a sustainability evaluation system for children’s food packaging, consisting of 5 core primary indicators and 11 secondary indicators. The CRITIC model was applied to process the sample data, determining the weights of the 11 indicator systems, including “Environmentally Friendly Material Selection”, “Material Safety”, and “Freshness and Protective Performance.” Subsequently, the study combined the MABAC model to rank the sustainability performance of 20 children’s food packaging design schemes (S1–S20). The ranking results showed that S1 was the best scheme, followed by S3 and S5. Based on the optimal S1 scheme, the study used children’s fruit puree packaging design as an example to carry out sustainable optimization design and presentation for children’s fruit puree packaging.

The study applied the CRITIC–MABAC evaluation model to the field of children’s food packaging design, proposing a new comprehensive evaluation and optimization framework. This research provides a new evaluation dimension for the sustainability design of children’s food packaging and offers practical optimization strategies for packaging design practice. However, the study has some limitations, such as the need to expand the sample selection, especially considering the different preferences of consumers from various cultures and regions. Additionally, children’s food preferences and habits at different stages of growth are areas that require further in-depth and detailed research. In response to these limitations, the next step in the research will be to further explore the quantitative evaluation and analysis of different consumer groups and behaviors in the field of food packaging. This will ensure that the sample data more comprehensively reflects consumer behavior and scientifically guides the design process while considering the diversity and uniqueness of children’s age groups. The goal is to promote the development of children’s food packaging towards a greener, more environmentally friendly, and safer direction.

## Figures and Tables

**Table 1:** Sustainability Evaluation Criteria for Children’s Food Packaging Design.

Primary Criteria	Secondary Criteria		
Eco-friendliness	Environmentally Friendly Material Selection (R1)	Minimalist Design (R2)	Lifecycle Environmental Impact (R3)
Safety	Material Safety (R4)	Child Protection Design (R5)	
Function	Ease of Use and Convenience (R6)	Freshness and Protective Performance (R7)	
Educational Value	Environmental Education Elements (R8)	Interactive Educational Function (R9)	
Economic Efficiency	Cost-Effectiveness (R10)	Market Competitiveness (R11)	

**Table 2:** Parent Consumer Sample Group for Children’s Food Packaging Design.

Category	Variable	Number of People	Percentage (%)	Cumulative Percentage (%)
Gender	Female	150	60	60
	Male	100	40	100
Age (years)	20–25	50	20	20
	26–31	100	40	60
	32–37	70	28	88
	38–43	30	12	100
Education Level	Middle School or Below	20	8	8
	High School	100	40	48
	Associate’s/College	120	48	96
	Master’s or Above	10	4	100
Occupation	Corporate Employee	80	32	32
	Government/Public Sector	60	24	56
	Freelancer	50	20	76
	Others	50	20	100
Household Monthly Income (CNY)	<2000	30	12	12
	2000–5000	120	48	96
	5000–8000	90	36	48
	>8000	60	24	100

**Table 3:** 9-Point Rating Scale Reference Table.

Impact Degree Rating	1	2	3	4	5	6	7	8	9
Impact Description	Negligible Impact	Minimal Impact	Very Low Impact	Low Impact	Moderate Impact	Moderately High Impact	High Impact	Very High Impact	Extreme Impact

**Table 4:** Sustainable Evaluation Indicator System and Design Element Conversion Overview Table.

No.	Indicator Layer	Design Layer
1	R1 (Environmentally Friendly Materials)	Fully biodegradable plastic, quick degradation
2	R2 (Minimalist Design)	Control illustration proportion, reduce packaging layers
3	R3 (Lifecycle)	Eco-friendly ink reduces VOC emissions, simplify packaging structure
4	R4 (Material Safety)	Food-grade materials, BPA-free
5	R5 (Child Protection)	Easy-open and leak-proof structure, clear labeling, and safety warnings
6	R6 (Ease of Use and Convenience)	Knob-style cap design, flat shape, easy to stack and store
7	R7 (Freshness and Protection)	High-barrier aluminum foil layer, resealable knob-style cap
8	R8 (Environmental Education)	Environmental-themed stories
9	R9 (Educational Interaction)	QR code linking to an environmental education platform
10	R10 (Cost-Effectiveness)	Cost allocation management and market positioning
11	R11 (Market Competitiveness)	Innovative brand building and market promotion

**Table 5:** Consumer Sustainability Scoring Table for Children’s Food Packaging Design Schemes.

	R1	R2	R3	R4	R5	R6	R7	R8	R9	R10	R11
S1	7	8	6	5	9	7	5	5	8	6	7
S2	6	7	5	5	8	6	4	4	7	7	6
S3	8	6	7	6	7	8	8	5	6	6	4
S4	5	9	4	7	6	5	6	6	9	5	8
S5	9	5	8	3	8	7	7	7	5	6	6
S6	6	8	7	4	7	6	5	5	8	7	9
S7	8	7	6	5	5	8	6	4	7	8	7
S8	8	6	5	6	7	9	4	6	7	5	6
S9	7	8	7	5	6	5	8	6	6	7	5
S10	9	5	6	7	8	7	6	4	5	8	3
S11	6	7	5	5	8	6	7	7	8	4	8
S12	8	6	7	6	7	8	5	5	6	9	4
S13	5	9	4	7	6	5	6	6	9	5	7
S14	9	5	8	3	5	7	7	7	5	6	4
S15	6	8	7	4	7	6	5	5	8	7	9
S16	8	7	6	6	5	8	6	4	7	8	7
S17	8	6	5	6	7	9	4	6	7	5	6
S18	7	8	7	5	6	5	8	6	6	7	5
S19	8	4	5	6	7	3	5	3	4	7	2
S20	6	7	5	5	8	6	7	7	8	4	8

**Table 6:** Standardized Values for Positive Indicators.

	R1	R2	R3	R4	R5	R6	R7	R8	R9	R10	R11
S1	0.50	0.80	0.50	0.50	1.00	0.67	0.25	0.50	0.80	0.40	0.71
S2	0.25	0.60	0.25	0.50	0.75	0.50	0.00	0.25	0.60	0.60	0.57
S3	0.75	0.40	0.75	0.75	0.50	0.83	1.00	0.50	0.40	0.40	0.29
S4	0.00	1.00	0.00	1.00	0.25	0.33	0.50	0.75	1.00	0.20	0.86
S5	1.00	0.20	1.00	0.00	0.75	0.67	0.75	1.00	0.20	0.40	0.57
S6	0.25	0.80	0.75	0.25	0.50	0.50	0.25	0.50	0.80	0.60	1.00
S7	0.75	0.60	0.50	0.50	0.00	0.83	0.50	0.25	0.60	0.80	0.71
S8	0.75	0.40	0.25	0.75	0.50	1.00	0.00	0.75	0.60	0.20	0.57
S9	0.50	0.80	0.75	0.50	0.25	0.33	1.00	0.75	0.40	0.60	0.43
S10	1.00	0.20	0.50	1.00	0.75	0.67	0.50	0.25	0.20	0.80	0.14
S11	0.25	0.60	0.25	0.50	0.75	0.50	0.75	1.00	0.80	0.00	0.86
S12	0.75	0.40	0.75	0.75	0.50	0.83	0.25	0.50	0.40	1.00	0.29
S13	0.00	1.00	0.00	1.00	0.25	0.33	0.50	0.75	1.00	0.20	0.71
S14	1.00	0.20	1.00	0.00	0.00	0.67	0.75	1.00	0.20	0.40	0.29
S15	0.25	0.80	0.75	0.25	0.50	0.50	0.25	0.50	0.80	0.60	1.00
S16	0.75	0.60	0.50	0.75	0.00	0.83	0.50	0.25	0.60	0.80	0.71
S17	0.75	0.40	0.25	0.75	0.50	1.00	0.00	0.75	0.60	0.20	0.57
S18	0.50	0.80	0.75	0.50	0.25	0.33	1.00	0.75	0.40	0.60	0.43
S19	0.75	0.00	0.25	0.75	0.50	0.00	0.25	0.00	0.00	0.60	0.00
S20	0.25	0.60	0.25	0.50	0.75	0.50	0.75	1.00	0.80	0.00	0.86

**Table 7:** Standardized Values for Negative Indicators.

	R1	R2	R3	R4	R5	R6	R7	R8	R9	R10	R11
S1	0.50	0.20	0.50	0.00	0.30	0.33	0.75	0.50	0.20	0.60	0.29
S2	0.75	0.40	0.75	0.50	0.25	0.50	1.00	0.75	0.40	0.40	0.43
S3	0.25	0.60	0.25	0.25	0.50	0.17	0.00	0.50	0.60	0.60	0.71
S4	1.00	0.00	1.00	0.00	0.75	0.67	0.50	0.25	0.00	0.80	0.14
S5	0.00	0.80	0.00	1.00	0.25	0.33	0.25	0.00	0.80	0.60	0.43
S6	0.75	0.20	0.25	0.75	0.50	0.50	0.75	0.50	0.20	0.40	0.00
S7	0.25	0.40	0.50	0.50	1.00	0.17	0.50	0.75	0.40	0.20	0.29
S8	0.25	0.60	0.75	0.25	0.50	0.00	1.00	0.25	0.40	0.80	0.43
S9	0.50	0.20	0.25	0.50	0.75	0.67	0.00	0.25	0.60	0.40	0.57
S10	0.00	0.80	0.50	0.00	0.25	0.33	0.50	0.75	0.80	0.20	0.86
S11	0.75	0.40	0.75	0.50	0.25	0.50	0.25	0.00	0.20	1.00	0.14
S12	0.25	0.60	0.25	0.25	0.50	0.17	0.75	0.50	0.60	0.00	0.71
S13	1.00	0.00	1.00	0.00	0.75	0.67	0.50	0.25	0.00	0.80	0.29
S14	0.00	0.80	0.00	1.00	1.00	0.33	0.25	0.00	0.80	0.60	0.71
S15	0.75	0.20	0.25	0.75	0.50	0.50	0.75	0.50	0.20	0.40	0.00
S16	0.25	0.40	0.50	0.25	1.00	0.17	0.50	0.75	0.40	0.20	0.29
S17	0.25	0.60	0.75	0.25	0.50	0.00	1.00	0.25	0.40	0.80	0.43
S18	0.50	0.20	0.25	0.50	0.75	0.67	0.00	0.25	0.60	0.40	0.57
S19	0.25	1.00	0.75	0.25	0.50	1.00	0.75	1.00	1.00	0.40	1.00
S20	0.75	0.40	0.75	0.50	0.25	0.50	0.25	0.00	0.20	1.00	0.14

**Table 8:** Correlation Between Sustainability Indicators.

R1	R2	R3	R4	R5	R6	R7	R8	R9	R10	R11
0.50	−0.24	0.20	−0.25	−0.01	1.00	−0.01	0.00	0.07	0.08	0.04
−0.83	1.00	−0.28	0.08	−0.15	−0.24	0.13	0.84	0.18	−0.18	0.71
0.54	−0.28	1.00	0.40	−0.11	0.20	0.13	−0.53	0.11	0.47	−0.22
−0.16	0.18	0.11	0.42	0.05	0.07	−0.35	0.24	1.00	−0.70	0.31
−0.09	−0.15	−0.11	−0.25	1.00	−0.01	−0.04	0.05	0.05	−0.27	0.05
1.00	−0.83	0.54	0.07	−0.09	0.50	−0.18	−0.83	−0.16	0.40	−0.67
−0.83	0.84	−0.53	−0.21	0.05	0.00	0.20	1.00	0.24	−0.45	0.87
−0.18	0.13	−0.70	−0.19	−0.04	−0.01	1.00	0.20	−0.35	0.00	−0.21
0.07	0.08	0.40	1.00	−0.25	−0.25	−0.19	−0.21	0.42	−0.11	−0.14
0.40	−0.18	0.47	−0.11	−0.27	0.08	0.00	−0.45	−0.70	1.00	−0.37
−0.67	0.71	−0.22	−0.14	0.05	0.04	−0.21	0.87	0.31	−0.37	1.00

**Table 9:** Sustainability Indicator Contrast, Conflict, Information Content, and Indicator Weights.

Indicators	Contrast	Conflict	Information Content	Weights
R1	0.3204	20.2434	6.4852	0.1048
R2	0.2798	18.7328	5.2424	0.0847
R3	0.3035	19.1307	5.8059	0.0938
R4	0.3292	19.1826	6.3154	0.1021
R5	0.2842	19.7801	5.6211	0.0908
R6	0.2564	18.6049	4.7694	0.0771
R7	0.2936	20.3671	5.9792	0.0966
R8	0.2798	18.8134	5.2649	0.0851
R9	0.2969	18.8362	5.5927	0.0904
R10	0.2774	20.1249	5.5825	0.0902
R11	0.2799	18.6425	5.2185	0.0843

**Table 10:** Total Distance to the Boundary Approximation Region (Ranking of Scheme Strengths and Weaknesses).

**Scheme**	**S1**	**S2**	**S3**	**S4**	**S** **5**	**S** **6**	**S** **7**	**S** **8**	**S** **9**	**S** **10**
${TD}_{i}$	0.215	0.079	0.210	0.158	0.208	0.181	0.170	0.148	0.190	0.167
**Scheme**	**S11**	**S12**	**S13**	**S14**	**S15**	**S16**	**S17**	**S18**	**S19**	**S20**
${TD}_{i}$	0.186	0.198	0.146	0.127	0.181	0.189	0.148	0.190	−0.05	0.186

## Data Availability

Data are available upon request.
